# Philadelphia Hospital

**Published:** 1839-01-19

**Authors:** 


### CLINICAL LECTURE.

### PHILADELPHIA HOSPITAL.

### SCROFULA.

*Saturday*, 15*th December*.—Dr. Jackson observed, that he should to-day present to the class several cases of patients affected with scrofula, and offer them some general views in relation to the character of this disease. This subject, said he, is one of considerable complications; and the great difficulty in medicine, as well as in all other sciences, consists in these complications. Rightly to understand any branch of science, we must break up the compound parts which constitute it, into their simple elements,—and the difficulty of doing this is, perhaps, greater in medicine than in any of the sister sciences. We are, however, in it, slowly advancing to the reduction of compound into simple elements; and, in the study of disease, it is chiefly by falling back upon our knowledge of anatomy and physiology, that this can be effected. Scrofula belongs to a class of affections, that can be understood only by a thorough knowledge of the organs and of the functions involved.

There is a natural group of diseases, separated from all others by distinctive and well-marked characters. They constitute a class by themselves,—and, from the organic element which is their essential base, may be termed lymphatic diseases. In this class we may place—1st. Scrofula, manifested most clearly in the lymphatic glands and vessels. 2d. Tubercular disease, consisting in a deposition of a specific kind of matter in various tissues. These two are often regarded as precisely the same; they are certainly different. Many patients have scrofula for years without tubercular disease; and numbers have tubercular diseases who have never manifested the slightest tendency to scrofula. 3d. White induration, hardening, or sclerosis:—this affection is chiefly limited to the derma, extends to the cellular tissue without gelatinous effusion; and, I suspect, is the affection in the lungs named by Laennec, tubercular infiltration, the tubercular character of which is very doubtful. 4th. Elephantiasis of the Arabs:—in this disease there is chronic enlargement, thickening and induration of the skin and cellular tissue, with gelatiniform effusion. 5th. Elephantiasis of the Greeks, originally a disease of the derma and cellular tissue, appearing in the form of solid tubercular tumours.

This group cannot be confounded with inflammations. They have no characters in common with inflammation. They are rarely acute, are chronic or slow in disposition. In their simple state there is no redness; red blood does not participate in the morbid action or its products; there is no increase of temperature; very seldom any alteration of sensibility; they never suppurate, or form proper pus. Thus, you perceive, that the characters of this group, distinctly defined, stand out in strong contrast with the equally well-marked characters of inflammatory diseases.

The seat of the lymphatic diseases is in the white tissues,—structures into which red blood does not penetrate, except when they are in a state of inflammation. They are nourished, formed out of lymph blood, and their mode of vitality, and their vital forces, are in relation with, and are sustained by lymph blood. While these tissues are subject to inflammation—when they change their character, becoming injected with red blood, and exhibit the ordinary signs and effects of inflammation—they are also subject to the pathological conditions before described, in which lymph blood and lymph capillaries alone are concerned in the structural alterations induced. It is a state, in its anatomical and physiological characters and elements, entirely dissimilar to inflammation.

The analysis may be extended further, and, embracing the general functions, throw additional light on some of the phenomena of scrofula, respecting which there prevails a diversity of opinion amongst authors.

The phenomena of life may be classed in three principal circles, to which there are others subordinate. The first are those of the nervous system, (the dynamic organs and phenomena of vitality,) to which the muscular system is subordinate. The second are those of the system of the mucous membranes and glandular system, which is an extension and ramification of the mucous membranes, (the plastic or formative organs and phenomena of vitality.)

The third are those of the circulatory apparatus, and of the blood. This circle is intermediate to the two first; it interlocks, as it were, with both, and sustains their activity. The blood is the element of life. The nervous or dynamic phenomena are in a state of immediate and unceasing relation with red, arterial, or oxygenated blood. It furnishes the elements of nervous energy or force, whatever that may be; and the interruption of its supply for four or five minutes is inevitable death, the extinction of the nervous or dynamic forces of the economy.

The blood, at the same time, furnishes to the various organs, the plastic elements, out of which the secretions and different structures of the economy are formed.

In this manner, the phenomena and functions of the two first circles are directly dependent on the quantity, the quality or composition, and the distribution or supply of this fluid.

Now, this vital fluid, while in the distributive apparatus of the circulatory system, (the heart and large blood-vessels,) presents the aspect of a homogeneous fluid; it is of a scarlet or of a purple hue. In the plastic or formative part of the system, (the capillaries,) it separates into two distinct kinds of blood,—red blood, and colourless or lymph blood. Thus, there are red or sanguine capillaries, and colourless or lymphatic capillaries.

These last are found in all the white tissues, those which are not nourished or formed of red blood, but which, in a healthy state, are penetrated only by colourless or lymph blood. The mode of vitality, the functions and arteries of those tissues, their capillaries and nutritive fluid, are totally dissimilar to the mode of vitality, the functions and arteries of the red tissues and organs composed of them, of the red capillaries and of the red blood. These differences prevail not only in health, but of necessity extend to disease.

While red capillaries and red blood belong almost exclusively to certain tissues, (muscular or contractile—some glandular, as liver and kidneys, and the spleen,)—and while colourless capillaries and lymph blood belong almost exclusively to other tissues, (cellular-serous, all the sclerous, nervous medulla,) there are other structures in which both are nearly equal, as the skin, and some of the mucous membranes. In these mixed structures, the proportion of the two kinds of capillaries is not a fixed one; it varies in different individuals. In some, the red predominate—the complexion is deep, dark, or sanguine. In others the lymph capillaries are ascendant, and the skin is fair, delicate, and pallid.

The subordinate circles are those of the sclerous and stratified tissues, (bones, cartilages, fibrous membranes:) the visceral envelopes, (serous and fibro-serous membranes:) and the external envelope or peripheric structure, (the skin.) This last is a mixed structure—the nervous is largely diffused in it—the mucous membranes participate, and the vascular in both its elements is also present.

This division of the phenomena or functions of life, and structure, is not hypothetical. It is the result of strict analysis, and is traced back to the first developement of the embryon. The formation of these three primary circles, is the movement to the formation of the embryon from the germ. The proligerous membrane of the egg, when fecundation has taken place, splits or divides into three layers. The superior, called by Pander and Burdach, serous, from its aspect, but which would be more properly named nervous, is that from which the spine, spinal marrow and brain are immediately formed. It is extended so as to enter into the formation of the skin, or its exterior. The lower is the mucous layer, and from it are formed the whole alimentary canal, the lungs and glands, and it contributes to the structure of the skin internally. Between the two is the vascular layer, a granular substance soon changing into blood, into the heart, becoming concrete in its walls and fluid in its centre and the great vessels. Thus the rudimentary form of the embryon is three different layers of plastic matter, which are developed and metamorphosed into three distinct circles of systems and functions, continuing throughout the future existence of the individual.

As these three circles exhibit specific phenomena in health, they present equally specific phenomena in disease. In pathology as in physiology, there are circles of phenomena. For the first circle we have, in their simple state, purely nervous disorders, (hallucinations of the senses, spasms, neuralgias.) For the second, the simple disorder of secretory and plastic functions of the nervous membranes and glands, (excess or absence of secretion, some forms of indigestion, constipation, &c.) For the third, connected with the red capillaries, red tissues, and red blood, there are inflammation and its results—(as the simple phlegmasiæ, pneumonitis, hepatitis, pleuritis, gastritis,) and connected with the white capillaries, white tissues and lymph blood, there are the lymphatic diseases, spoken of already (scrofula, tubercular disease, and the others named before.)

Now the above elementary diseases belonging to the primary tissues and organs, and affecting the especial functions already pointed out, may occur singly, locally, generally, or they may take place in combination; nervous, sanguine inflammatory, or lymphatic disease may exist, each alone; or nervous affection and inflammation may be conjoined; or nervous and lymphatic may concur, or lymphatic disease and inflammation be associated as is seen in some cases of scrofula—and especially in many ulcerative and eruptive diseases of the skin.

If, now, to the above simple and primary disorders, we add those produced by specific poisons, animal, vegetable, or mineral; and again, conjoin those resulting from specific contagions, causing eruptive fevers, small pox, scarlatina, &c.; and again, to the above unite those produced by aerial, tellurial and miasmatic influences, as influenzas, wide-spread epidemics, spasmodic cholera, fevers, epidemic and endemic; and keep in view the consequences on all the functions and vital phenomena of a depraved condition of the blood that may occur in disease; we shall possess the elements of nearly all the pathological conditions to which the human economy is subject.

We have thus broken down the pathological conditions into their primary elements, and remembering that these elements unite in every possible proportion, by knowing them in their separate state, we can recognise them in their combination, and assign to each phenomenon or symptom of a complex affection its proper source. When the simple is well known, the compound cannot be misunderstood.

Having by this analytical process, attempted to break up the phenomena of disease, and to reduce them to the expression of their simple states, the views I entertain with respect to scrofula, will probably be more clearly understood.

Scrofula, as was before stated, belongs to the class of lymphatic diseases, and I place it first on that list, as being the most frequent in occurrence.

It presents itself under different forms, constituting different species or varieties:

1st. As affecting the lymphatic glands,—Glandular Scrofula.

2d. As affecting the cartilages and ligaments of the joints,—Articular Scrofula.

3d. As attacking the periosteum, and thus involving the bones,—Periosteal and Osseous Scrofula.

4th. As affecting the skin and some of the more exterior mucous membranes,—Cutaneous Scrofula.

Scrofula not only occurs in the above forms, which are its purer or simpler expression in particular tissues, but it is capable of being excited by, and becoming associated with other affections, constituting a complicated or hybrid disease. For instance, the exciting cause of scrofula may be syphilitic ulcers or buboes, and being developed in the seat and tissue affected with syphilis, a modified disease is produced, generally of very inveterate character, and requiring great prudence in its treatment. This form may be termed syphilitic scrofula.

Mercury administered without reflection and and discrimination, often induces constitutional and local affections, that, no doubt, have often been mistaken for syphilis itself. The throat, the periosteum, the fibrous tissue generally, and the skin, are liable to the mercurial forms of disease, appearing as inflammations, swellings, pains, ulcers, and eruptions. In persons of the lymphatic temperament, and scrofulous diathesis, the mercurial diseases will become exciting causes of scrofula, and as in syphilis, the two become conjoined. Cases of this character I have uniformly found intractable, entailing great suffering, and difficult in treatment. It may be termed mercurial scrofula.

The specific cutaneous diseases of an inflammatory and eruptive character, also excite scrofulous disease, which may then run on in conjunction. Many of them appear, in their proper character, to be an association of inflammation and lymphatic disease. The existence of sanguine and lymphatic capillaries, largely distributed in the skin, and in nearly equal proportions, renders this combination not only possible but probable. It is theoretically demonstrated, and from the peculiar characters, products in the forms of secretions, crusts, squamæ, &c., and treatment of these affections, would appear to be equally demonstrated by the phenomena of the diseases themselves.

Such are the various forms and combinations, in which, it appears to me, I have met with scrofula: at least, from an attentive observation of numerous different cases that have fallen under my notice, and reflection on their peculiar characters, they have struck me as presenting the forms in which I have arranged them.

It is not my intention to present to you a history of the different lymphatic diseases, or even to enter upon a discussion of the different forms of scrofula. An elaborate work would be required, or a whole course of lectures devoted to that subject alone. I shall merely direct your attention to the first form of scrofula—the glandular—as the one most frequently occurring in practice.

The seat of glandular scrofula is the lymphatic glands. These are small vascular bodies connected with the lymphatic vessels. They appear, in fact, to be merely congeries of those vessels, connected by cellular tissue, and exhibiting in their centre the appearance of cells. The lymphatics are properly the veins of the lymph blood, returning the lymph from the white tissues in which it has been distributed, which it has nourished and supported, precisely as the veins are the returning vessels of the red blood from the red or coloured tissues. Their course is interrupted by their formation or arrangement into the glands, so called. What is the office of these glands, is entirely unknown. Many hypotheses have been formed respecting their uses, but as they are mere conjectures, "the coinage of the brain," it is needless to notice them.

These bodies are more numerous in some positions than others. The neck, the arm-pits, and the groins, are the points where they are most abundant, externally. In the mesentery, the root of the lungs, and along the spinal column, in the loins and back, they are found, internally, in the greatest profusion.

Glandular scrofula, is thus divisible into two varieties, the first, external, as it affects the glands externally: the second, internal, as the internal glands may be the seat of the disease.

External glandular scrofula most frequently is seen in the glands of the neck; in those of the groin, it is next as to frequency; and in those of the arm-pit its occurrence is more rare.

Internal glandular scrofula has its most frequent location in the glands, though this may be disputed: it is frequent also in the mesenteric glands, especially of children, and seldom affects the others. Internal scrofula is, most generally, secondary to affections of the mucous membranes, with which they are connected, through the lymphatics they belong to, which have their origin in those membranes.

There are but few cases of scrofula, at present, in the wards, and they do not present all of the characters of the disease.

In this black, you perceive a number of tumours along the neck, about the size of a walnut, or that of a small egg. They have only recently, that is within three or four months past, made their appearance with him, and the disease was preceded by syphilis, for which he entered the house—he has been cured of that disease, and you now see that scrofula has succeeded to it.

In this case, also a black, the disease has reached its termination. You notice his neck is covered with cicatrices of a peculiar character. They are rough, punctured, on some are ridges or elevations, and the skin around them, as you can feel, possesses a remarkable hardness or induration. This last is not a common character of scrofulous cicatrices. It is, in a slight degree, the induration, which I have mentioned as one of the forms of lymphatic disease. The reuniting of the skin here will give you some idea of that form of disease.

Here is a young child, and you notice numerous small tumours along its neck and under the jaws. They are firm and insensible. They are less now than they were some time past. They are diminishing under the treatment, and working on to a resolution of the disease. In this case, as he has just informed you, the disease occupied from its commencement to its termination, a period of four years. In this woman you can see some small ulcers in the neck; and on examining them nearer, you will find the skin for some distance is loose and detached from the parts beneath it, by the destruction of the cellular tissue. The disease is here coming to a close; the glands are destroyed; the diseased parts have been removed by the process of disease, and cicatrization is about commencing. The skin here cannot participate in the act. You can see that it is a thin, detached layer, having no connection beneath it. The dull red colour of this loosened skin is not inflammation: it is a passive state, arising from its want of vascular connection beneath it, and the consequent want of an active circulation.

The glands in the axilla of this woman are enlarged and indurated. They have not yet softened; and are in the first period of the same disease, which has reached its last in the neck.

These are all the cases that can now be exhibited to illustrate to you the exterior characters of the disease. You find in all of them that the lymphatic glands are the seat of the affection. In none of these cases has it extended beyond the glands, with the exception of the skin covering or immediately around them in those that ulcerated. In the others the skin remains perfectly healthy.

